# Correction: The long non-coding RNA Cerox1 is a post transcriptional regulator of mitochondrial complex I catalytic activity

**DOI:** 10.7554/eLife.50980

**Published:** 2019-08-12

**Authors:** Tamara M Sirey, Kenny Roberts, Wilfried Haerty, Oscar Bedoya-Reina, Sebastian Rogatti-Granados, Jennifer Y Tan, Nick Li, Lisa C Heather, Roderick N Carter, Sarah Cooper, Andrew J Finch, Jimi Wills, Nicholas M Morton, Ana Claudia Marques, Chris P Ponting

Sirey TM, Roberts K, Haerty W, Bedoya-Reina O, Rogatti-Granados S, Tan JY, Li N, Heather LC, Carter RN, Cooper S, Finch AJ, Wills J, Morton NM, Marques AC, Ponting CP. 2019. The long non-coding RNA *Cerox1* is a post transcriptional regulator of mitochondrial complex I catalytic activity. *eLife* **8**:e45051. doi: 10.7554/eLife.45051.

Published 2, May 2019

Since publication of our original article we noticed that our representation of the findings of Delás et al. (2019) was incorrect, specifically with respect to the effect of *Cerox1* knock-down in haematopoietic stem cells. We apologise for our error, and have corrected the article in four places.

(1) Original text (Results section):

Finally, it has recently been demonstrated in a mouse model of haematopoietic lineage differentiation that *Cerox1* depletion impairs the contributions of stem and progenitor cells to haematopoiesis.

Corrected text:

Finally, it has recently been demonstrated in a mouse model of haematopoietic lineage differentiation that *Cerox1* depletion may impair the contributions of stem cell or B cell differentiation to haematopoiesis.

(2) Original text (Discussion):

and in haematopoiesis where depletion of *Cerox1* results in decreased expression of OXPHOS components and in defects during differentiation of the committed myeloid progenitor population

Corrected text:

and in haematopoiesis where depletion of *Cerox1* results in B cell depletion or myeloid enrichment

(3) Original text (Response to Reviewers):

The following line has been removed, as this was not relevant to the lncRNA *CEROX1.*

Others have more recently verified the cytoplasmic localisation of *CEROX1* in human leukaemia cells (Delas et al., 2018)

(4) Original text (response to reviewers):

In a mouse model of haematopoietic lineage differentiation depletion of *Cerox1* (in this instance called Spehd) was shown to impair multi-lineage differentiation (Delas et al., 2018).

Corrected text:

In a mouse model of haematopoietic lineage differentiation depletion of lncRNAs highly expressed in haematopoietic stem cells leads to a deficit in B-lymphocyte differentiation (*Cerox1,* in this instance called lnc6689; Delás et al., 2019).

The article has been corrected accordingly.

