# Correction: The long non-coding RNA *Cerox1* is a post transcriptional regulator of mitochondrial complex I catalytic activity

**DOI:** 10.7554/eLife.106198

**Published:** 2025-02-03

**Authors:** Tamara M Sirey, Kenny Roberts, Wilfried Haerty, Oscar Bedoya-Reina, Sebastian Rogatti-Granados, Jennifer Y Tan, Nick Li, Lisa C Heather, Roderick N Carter, Sarah Cooper, Andrew J Finch, Jimi Wills, Nicholas M Morton, Ana Claudia Marques, Chris P Ponting

**Keywords:** Human, Mouse

Sirey TM, Roberts K, Haerty W, Bedoya-Reina O, Rogatti-Granados S, Tan JY, Li N, Heather LC, Carter RN, Cooper S, Finch AJ, Wills J, Morton NM, Marques AC, Ponting CP. 2019. The long non-coding RNA Cerox1 is a post transcriptional regulator of mitochondrial complex I catalytic activity. *eLife* **8**:e45051. doi: 10.7554/eLife.45051.

Published 2 May 2019

A reader kindly alerted us to errors in Figure 7 panels D and F in the original publication. We then realised that data sets were wrongly included at the final stage of manuscript preparation, when we substituted the original submission’s barplots with box plots showing data points. These errors affected the 3 Complex I panels of Figure 7D/F. We have now replaced these panels, and their data, using the data used for the original submission.

Upon further review, two further inconsistencies were evident. (i) Data for Complex III (Figure 7D) was for a singlicate data set, not data from duplicates as stated in the Methods. We now substitute this with a duplicate data set, obtained contemporaneously with all other data in this Figure, which – as with the published singlicate data set – shows a significant increase in Complex III activity following *CEROX1* overexpression, relative to control (*P*=2.70 × 10^–7^). (ii) The effect of *hCEROX1* overexpression on Citrate synthase activity in mouse N2A cells was significant (*P*=1.6 × 10^–3^) and not, as indicated in the publication, “ns” – not significant.

The manuscript’s results and conclusions remain unaffected by these errors. This is because its findings and text were based on the original data (evident in the original submission’s barplots), not on the data erroneously included during the revision. No corrections to the text were needed.

The corrected Figure 7 is shown here:

**Figure 1:**
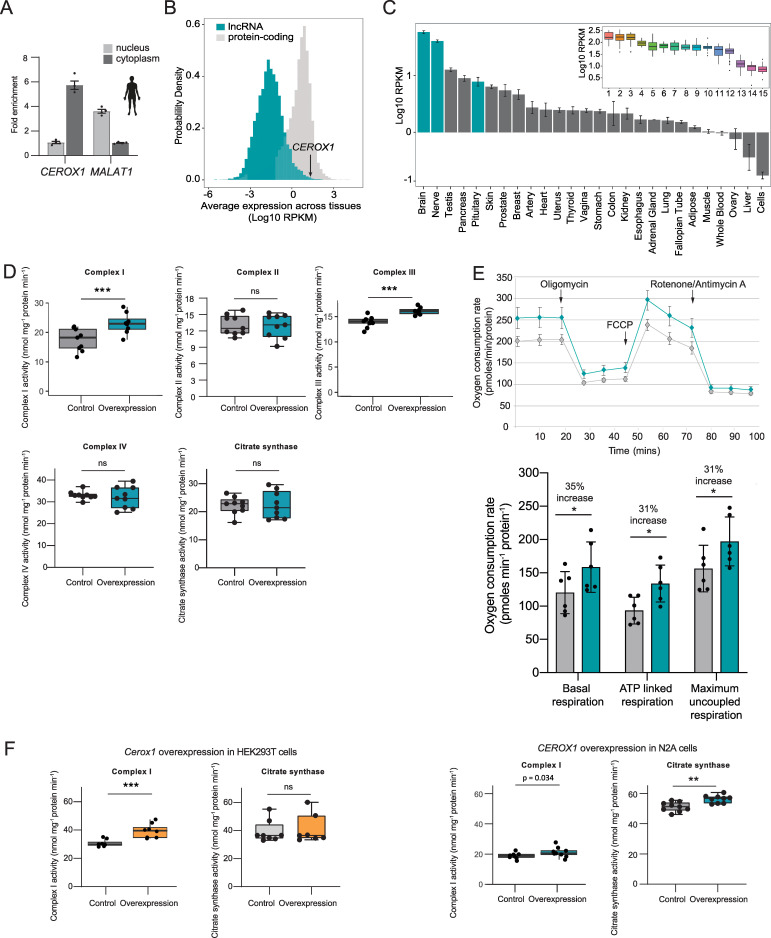


The originally published Figure 7 is shown for reference:

**Figure 2:**
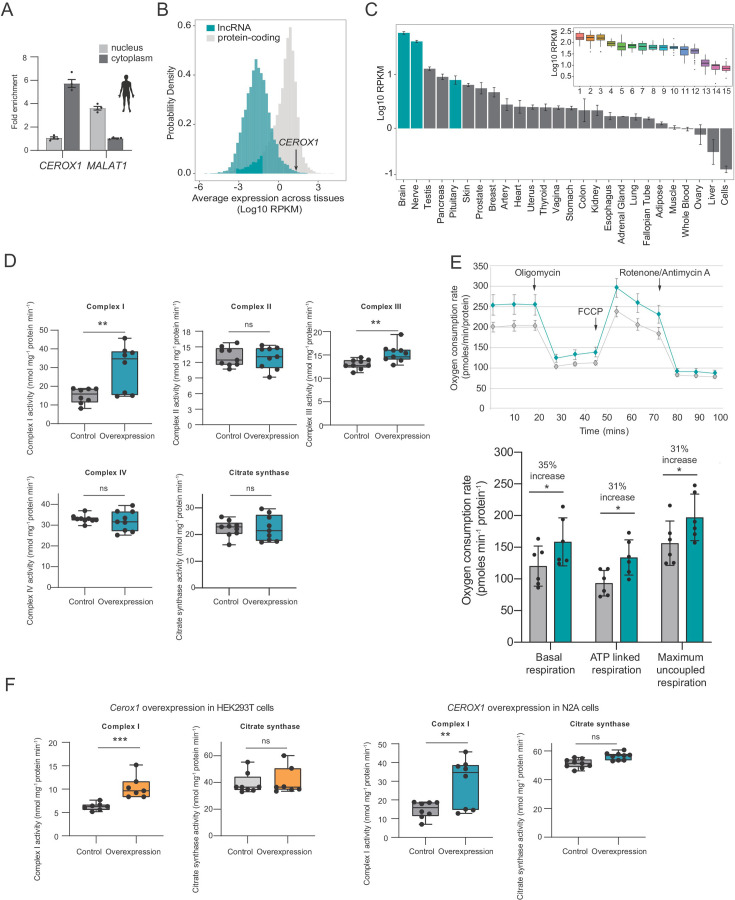


The source data for Figure 7D (Figure 7—source data 1) and Figure 7F (Figure 7—source data 3) have been corrected. The article has been corrected accordingly.

