# Supplementary material for: The long non-coding RNA Cerox1 is a post transcriptional regulator of mitochondrial complex I catalytic activity
Source: eLife. 2019 May 2;8:e45051. doi: 10.7554/eLife.45051 (PMC6542586; doi:10.7554/eLife.45051)
Supplement: Transparent reporting form [file elife-45051-transrepform.docx]

***eLife’s* transparent reporting form**

We encourage authors to provide detailed information *within their submission* to facilitate the interpretation and replication of experiments. Authors can upload supporting documentation to indicate the use of appropriate reporting guidelines for health-related research (see [EQUATOR Network](http://www.equator-network.org/%20)), life science research (see the [BioSharing Information Resource](https://biosharing.org/)), or the [ARRIVE guidelines](http://www.plosbiology.org/article/info:doi/10.1371/journal.pbio.1000412) for reporting work involving animal research. Where applicable, authors should refer to any relevant reporting standards documents in this form.

If you have any questions, please consult our Journal Policies and/or contact us: [editorial@elifesciences.org](mailto:editorial@elifesciences.org).

**Sample-size estimation**

- You should state whether an appropriate sample size was computed when the study was being designed
- You should state the statistical method of sample size computation and any required assumptions
- If no explicit power analysis was used, you should describe how you decided what sample (replicate) size (number) to use

Please outline where this information can be found within the submission (e.g., sections or figure legends), or explain why this information doesn’t apply to your submission:

No explicit power calculations were used to calculate sample sizes except in the case of the specific CI, CII, CIII, CIV and CS enzyme assays (analysis shown in Figures 3A, 3C, 5D, 6F, 7D, 7E – results section). For quantitative PCR gene expression analysis of transfected cells, data was collected from 3 biological replicates of control and experimental samples, and technical replicates for each gene being assayed were performed in triplicate on each quantitative PCR plates (analysis shown in results section figures 2C, 5A, 5C, 5E, 6A, 6B, 6E). This approach is well-accepted in molecular biology. Transfections were performed with either 3-4 (7 total) (results section figure 2D) or 6 (results section figure 4B) for experiments harvested for western blots. The protein carbonylation experiment (figure 4B) was performed with greater replication as advised by the manufacturer. Cellular fractionations were performed on 5 biological replicates (results section figures 1F) or 4 biological replicates (results section figure 7A). Replication for the Seahorse bioanalyzer experiments was determined by the availability of 20 wells per plate (4 wells are allocated as background) designating 5 replicates for the overexpression and silencing conditions and their respective controls (results section figures 3B and 3D). Assays performed in 96 well plates were designed with either 12 replicates (results section figure 4A) or 6 replicates (results section figure 4C) to minimise between plate effects.

**Replicates**

- You should report how often each experiment was performed
- You should include a definition of biological versus technical replication
- The data obtained should be provided and sufficient information should be provided to indicate the number of independent biological and/or technical replicates
- If you encountered any outliers, you should describe how these were handled
- Criteria for exclusion/inclusion of data should be clearly stated
- High-throughput sequence data should be uploaded before submission, with a private link for reviewers provided (these are available from both GEO and ArrayExpress)

Please outline where this information can be found within the submission (e.g., sections or figure legends), or explain why this information doesn’t apply to your submission:

For transfection experiments where the downstream analysis was quantitative PCR we collected data from 3 independent transfections for both control and experimental conditions, we define these as biological replicates. For gene expression analysis each biological replicate was technically replicated in triplicate for each gene assayed to determine a measure of technical variation. Data was excluded in the case of obvious technical error (>0.5 Ct difference between technical replicates) or erroneous melt curve.

- Analysis shown in Figure 1F - Results section
- Analysis shown in Figures 2C,2D - Results section
- Analysis shown in Figures 3A, 3B, 3C,3D - Results section
- Analysis shown in Figures 4A,4B,4C - Results section
- Analysis shown in Figures 5A,5C,5D,5E,5F - Results section
- Analysis shown in Figures 6A,6B,6D,6E,6F,6G - Results section
- Analysis shown in figures 7A,7D,7E,7F – Results section

**Statistical reporting**

- Statistical analysis methods should be described and justified
- Raw data should be presented in figures whenever informative to do so (typically when N per group is less than 10)
- For each experiment, you should identify the statistical tests used, exact values of N, definitions of center, methods of multiple test correction, and dispersion and precision measures (e.g., mean, median, SD, SEM, confidence intervals; and, for the major substantive results, a measure of effect size (e.g., Pearson's r, Cohen's d)
- Report exact p-values wherever possible alongside the summary statistics and 95% confidence intervals. These should be reported for all key questions and not only when the p-value is less than 0.05.

Please outline where this information can be found within the submission (e.g., sections or figure legends), or explain why this information doesn’t apply to your submission:

N values, statistical analysis methods and significance are reported in each figure legend or within the main text.

- Analysis shown in Figure 1E - Results and Methods sections
- Analysis shown in Figure 2A - Results and Methods sections
- Analysis shown in Figure 2D - Results section
- Analysis shown in Figure 2E – Results and Methods sections
- Analysis shown in Figure 3A and 3C - Results section
- Analysis shown in Figure 3B and 3D - Results section
- Analysis shown in Figure 4A,B,C - Results section
- Analysis shown in Figure 5D - Results section
- Analysis shown in Figure 6A,6B,6D,6F,6G - Results sections
- Analysis shown in Figure 7C - Results and Methods sections
- Analysis shown in Figures 7D, 7E, 7F – Results section

(For large datasets, or papers with a very large number of statistical tests, you may upload a single table file with tests, Ns, etc., with reference to sections in the manuscript.)

**Group allocation**

- Indicate how samples were allocated into experimental groups (in the case of clinical studies, please specify allocation to treatment method); if randomization was used, please also state if restricted randomization was applied
- Indicate if masking was used during group allocation, data collection and/or data analysis

Please outline where this information can be found within the submission (e.g., sections or figure legends), or explain why this information doesn’t apply to your submission:

Samples were allocated into groups based on treatment as indicated in the figure legends.

**Additional data files (“source data”)**

- We encourage you to upload relevant additional data files, such as numerical data that are represented as a graph in a figure, or as a summary table
- Where provided, these should be in the most useful format, and they can be uploaded as “Source data” files linked to a main figure or table
- Include model definition files including the full list of parameters used
- Include code used for data analysis (e.g., R, MatLab)
- Avoid stating that data files are “available upon request”

Please indicate the figures or tables for which source data files have been provided:

- No additional data files have been uploaded.
